# Fucoidan present in brown algae induces apoptosis of human colon cancer cells

**DOI:** 10.1186/1471-230X-10-96

**Published:** 2010-08-22

**Authors:** Eun Ji Kim, So Young Park, Jae-Yong Lee, Jung Han Yoon Park

**Affiliations:** 1 Department of Food Science and Nutrition, Hallym University, Chuncheon, 200-702, Korea; 2 Center for Efficacy Assessment and Development of Functional Foods and Drugs, Hallym University, Chuncheon, 200-702, Korea; 3 Department of Biochemistry, College of Medicine, Hallym University, Chuncheon, 200-702, Korea; 4 Medical & Bio-Materials Research Center, Hallym University, Chuncheon, 200-702, Korea

## Abstract

**Background:**

Fucoidan is a sulfated polysaccharide found in brown algae; it has been shown to exhibit a number of biological effects, including anti-tumor effects. In this study, we evaluated the effects of fucoidan on apoptosis in HT-29 and HCT116 human colon cancer cells.

**Methods:**

HT-29 and HCT116 cells were cultured with various concentrations of fucoidan (0 - 20 μg/mL). Apoptosis was assayed via Hoechst staining and Annexin V staining followed by flow cytometric analysis. Western blot analyses and JC-1 staining were conducted to determine the levels of apoptosis-regulating proteins and mitochondrial membrane permeability, respectively.

**Results:**

Fucoidan induced substantial reductions in viable cell numbers and apoptosis of HT-29 and HCT116 cells in a dose-dependent manner. In HT-29 cells, fucoidan also increased the levels of cleaved caspases-8, -9, -7, and -3, and cleaved poly (ADP-ribose) polymerase (PARP) levels. The levels of the X-linked inhibitor of apoptosis protein and survivin were attenuated in the fucoidan-treated cells. Fucoidan was also shown to enhance mitochondrial membrane permeability, as well as the cytochrome c and Smac/Diablo release from the mitochondria. Fucoidan increased the levels of the Bak and truncated Bid proteins, but reduced the levels of Mcl-1. Additionally, fucoidan increased the levels of the tumor necrosis factor-related apoptosis-inducing ligand, Fas and death receptor 5 proteins. The caspase-8 and -9 inhibitors Z-IETD-FMK and Z-LEHD-FMK induced a reduction in fucoidan-mediated apoptosis. Caspase-8 inhibitor inhibited the fucoidan-induced cleavage of Bid, caspases-9 and -3, and PARP.

**Conclusion:**

The findings of this study indicate that fucoidan induces apoptosis in HT-29 and HCT116 human colon cancer cells, and that this phenomenon is mediated via both the death receptor-mediated and mitochondria-mediated apoptotic pathways. These results suggest that fucoidan may prove useful in the development of a colon cancer-preventive protocol.

## Background

Colorectal cancer is one of the most prevalent cancers in the United States and is the second-most-frequent cause of cancer-related mortality [1]. Additionally, the worldwide incidence rates of this cancer have been increasing steadily in recent years. Although early-stage colorectal cancer can be successfully treated surgically, advanced-stage colorectal cancer frequently recurs and becomes fatal, even in patients receiving combination chemotherapy [2]. Chemotherapeutic agents such as cisplatin are routinely used in the treatment of advanced-stage colorectal cancer, but provide only minimal survival benefits, due to several factors--including drug resistance, side effects, and toxicity [3,4]. Recently, the development of cancer chemoprevention protocols employing natural or synthetic agents for the prevention or suppression of progression to invasive cancer has been recognized as a field with enormous potential to reduce cancer burden [5]. Therefore, there is an urgent need for novel chemopreventive agents with minimal or no side effects and toxicities. In recent years, bioactive compounds derived from natural sources have become the focus of a substantial amount of attention from researchers seeking to develop chemopreventive agents, due primarily to the potential cancer-preventive and/or therapeutic activities of many of these compounds at non-toxic levels. However, continued research into the action mechanisms of such compounds will be necessary for credible assessments of the cancer chemopreventive qualities of these bioactive food components.

Fucoidan is a complex sulfated polysaccharide that is found in the cell walls of several edible brown algae, including *Fucus vesiculosus*. The structures and compositions of fucoidan vary among different brown seaweed species, but generally the compound consists primarily of L-fucose and sulfate, along with small quantities of D-galactose, D-mannose, D-xylose, and uronic acid [6-8]. Many previous reports have shown that fucoidan exerts anti-bacterial [9], anti-viral [10], anti-coagulant [11], antioxidant [12], anti-inflammatory [11,13], and immunomodulatory effects [9,14]. There have also been a variety of studies addressing the anticarcinogenic effects of fucoidan. In previous *in vivo *studies conducted using xenograft models, fucoidan has been reported to suppress the growth of Ehrlich ascites carcinoma [15,16] and Lewis lung adenocarcinoma [17], and has also been shown to inhibit the metastasis of Lewis lung adenocarcinoma [17] and 13762 MAT rat mammary adenocarcinoma [18]. The findings of previous *in vitro *studies have demonstrated that fucoidan inhibits the growth of non-small-cell bronchopulmonary carcinoma NSCLC-N6 cells [19] and human lymphoma HS-Sultan cells [20], and also inhibits the invasion of HT1080 human fibrosarcoma cells and the angiogenic activity of HeLa human uterine carcinoma cells [21]. However, to the best of our knowledge, the effects of fucoidan on the growth of colon cancer cells and its underlying mechanisms have yet to be determined in detail.

The inhibition of apoptosis, a universal and efficient cellular suicide pathway, is known as one of the hallmark characteristics of cancer [22]. The transformation of colorectal epithelium to carcinoma, in particular, is associated with a progressive inhibition of apoptosis. The inhibition of apoptosis in colorectal cancer contributes to tumor growth, promotes neoplastic progression, and confers resistance to cytotoxic anticancer agents [23]. Therefore, bioactive compounds with the ability to induce apoptosis in cancer cells can be employed as cancer chemopreventive and/or chemotherapeutic agents. Apoptosis occurs via two principal pathways: namely, the mitochondria-mediated and death receptor-mediated pathways. The receptor-mediated pathway is triggered by the binding of death-inducing ligands to cell surface receptors. The mitochondria-mediated pathway is triggered by a variety of apoptotic stimuli, which converge at the mitochondria, leading to the release of cytochrome c from the mitochondria to the cytoplasm. The two apoptosis pathways converge on caspase-3 and subsequently on other proteases and nucleases that drive the terminal events of apoptosis. These apoptosis pathways are tightly controlled by a variety of regulators, including the caspases, Bcl-2 family proteins, and the inhibitor of apoptosis protein (IAP) family [24,25].

The principal objective of this study was to determine whether fucoidan inhibits the growth of colon cancer cells, and to determine the mechanisms relevant to this effect. We determined that fucoidan induces apoptosis in HT-29 human colon cancer cells via both death receptor-mediated and mitochondria-mediated pathways.

## Methods

### Materials

The reagents employed in this study were purchased from the indicated suppliers: 3-[4,5-dimethylthiazol-2-yl]-2,5-diphenyltetrazolium bromide (MTT), biobenzimide H 33258 (Hoechst 33258), Z-IETD-FMK, Z-LEHD-FMK, 5,5',6,6'-tetrachloro-1,1',3,3'-tetraethyl-imidacarbocyanine iodide (JC-1), anti-β-actin antibody, and anti-α-tubulin antibody (Sigma-Aldrich Co.); Dulbecco's Modified Eagle's Medium/Ham's F-12 nutrient mixture (DMEM/F-12) (Gibco BRL, Gaithersburg, MD, USA); fetal bovine serum (FBS) (Cambrex Bio Technology, Walkersville, MD, USA); a horseradish peroxidase (HRP)-conjugated anti-rabbit, anti-goat, and anti-mouse IgG (Amersham Biosciences, Arlington Heights, IL, USA); antibodies against cleaved caspase-3, cleaved caspase-7, cleaved caspase-9, cleaved poly (ADP-ribose) polymerase (PARP), caspase-8, Bid, survivin, and X-linked inhibitor of apoptosis protein (XIAP) (Cell Signaling Technology, Beverly, MA, USA); phycoerythrin-conjugated Annexin V (PE-Annexin V), 7-amino-actinomycin D (7-AAD), and antibodies against cytochrome c and tumor necrosis factor-related apoptosis-inducing ligand (TRAIL) (BD Pharmingen, Franklin Lake, NJ, USA); antibodies against Bcl-2, Bax, Fas, Fas ligand (FasL), Smac/Diablo, and heat shock protein (HSP) 60 (Santa Cruz Biotechnology, Santa Cruz, CA, USA); antibodies against death receptor (DR) 4 and 5 (Imgenex, San Diego, CA, USA). Where not noted otherwise, all other materials were acquired from Sigma-Aldrich Co.

### Cell culture and cell viability assay

HT-29 and HCT116 human colon cancer cells and FHC human normal colon epithelial cells were obtained from the American Type Culture Collection (Manassas, VA, USA). HT-29 and HCT116 cells were maintained in DMEM/F12 containing 100 mL/L of FBS with 100,000 U/L of penicillin and 100,000 mg/L of streptomycin. FHC cells were maintained in DMEM/F12 supplemented with 100 mL/L of FBS, 10 μg/L of cholera toxin, 5 mg/L of insulin, 5 mg/L of transferrin, 100 μg/L of hydrocortisone, 100,000 U/L of penicillin, and 100,000 mg/L of streptomycin. In an effort to characterize the effects of fucoidan on cell growth, we plated cells in 24-well plates with DMEM/F-12 containing 100 mL/L of FBS. Prior to fucoidan treatment, the cell monolayers were rinsed and serum-deprived for 24 h with DMEM/F-12 containing 10 mL/L of charcoal-stripped FBS (serum-deprivation medium). Following serum-deprivation, the monolayers were treated with various concentrations (0, 5, 10, 20 μg/mL) of fucoidan in serum-deprivation medium for 24, 48 or 72 h. Viable cell numbers were estimated via an MTT assay, as described previously [26]. The fucoidan (Sigma) was prepared from *Fucus vesiculosus *via a modified version of the method described by Black *et al. *[27] and a crude polysaccharide composed predominantly (> 95%) of sulfated fucose. We employed the serum deprivation medium containing 10 mL/L of charcoal-stripped FBS in order to minimize the possible effects of various growth factors and phytochemicals in the FBS.

### Detection of the morphological changes due to apoptosis

In order to determine whether or not fucoidan induces chromatin condensation and fragmentation, both of which are recognized morphological features of apoptosis, HT-29 cells were plated on cell culture coverslips with DMEM/F-12 containing 100 mL/L of FBS. One day later, the cells were serum-deprived with serum-deprivation medium for 24 h. After serum deprivation, the cells were incubated for 72 h in serum-deprivation medium containing 0 or 20 μg/mL of fucoidan. The cells were stained with 10 mg/L of Hoechst 33258 dye and then examined via fluorescent microscopy, as previously described [28].

### Quantification of apoptotic cells

HT-29 and HCT116 cells were plated in 24-well plates with DMEM/F-12 containing 100 mL/L of FBS. One day later, the cells were serum-deprived with serum-deprivation medium for 24 h. After serum deprivation, the cells were incubated for 72 h in serum-deprivation medium containing 0, 5, 10, or 20 μg/mL of fucoidan. The numbers of early apoptotic cells were estimated via PE-Annexin V and 7-AAD staining as previously described [26]. After staining, we performed flow cytometry using a FACScan™system (Becton Dickinson, Franklin Lake, NJ, USA), and then the data were analyzed using ModFit V.1.2. Software (Becton Dickinson).

### Flow cytometric measurement of mitochondrial membrane potential

HT-29 cells were plated in 24-well plates with DMEM/F-12 containing 100 mL/L of FBS. One day later, the cells were serum-deprived with serum-deprivation medium for 24 h. After serum deprivation, the cells were incubated for 48 h in serum-deprivation medium containing 0, 5, 10, or 20 μg/mL of fucoidan. We determined the mitochondrial membrane potential using the dual emission dye, JC-1, in accordance with the method described previously by Jung *et al. *[29]. After staining the cells with JC-1, the numbers of cells exhibiting green and red fluorescence were quantified via flow cytometry using FACScan™, and then the data were analyzed with ModFit V.1.2. software.

### Western blot analysis

HT-29, HCT116, and FHC cells were plated in 100 mm dishes with DMEM/F-12 containing 100 mL/L of FBS. The next day, the cells were serum-deprived for 24 h with serum-deprivation medium. After serum deprivation, the cells were incubated in serum-deprivation medium containing 0, 5, 10, or 20 μg/mL of fucoidan for 36, 48, or 60 h. The total cell lysates were then prepared as previously described [30]. Cytosolic proteins were separated in accordance with the method described by Eguchi *et al. *[31]. We determined the protein contents in the total cell lysates and cytoplasmic fractions using a BCA protein assay kit (Pierce, Rockford, IL, USA). The proteins of the total cell lysates and cytoplasmic fractions were subsequently resolved on a sodium dodecyl sulfate (SDS) - 4% to 20% or 10% to 20% polyacrylamide gel, and then transferred onto polyvinylidene fluoride membranes (Millipore, Bedford, MA, USA). Western blot analyses were conducted as previously described [30]. We detected the signals on the basis of enhanced chemiluminescence using SuperSignal^® ^West Dura Extended Duration Substrate (Pierce). The relative abundance of each band was quantified via the Bio-profile Bio-1 D application (Vilber-Lourmat, Marine la Vallee, France), and the expression levels were normalized to β-actin.

### Statistical analysis

The results were expressed as the means ± SEM, and analyzed via ANOVA. Differences among the treatment groups were analyzed via Duncan's multiple-range tests using the SAS system for Windows V 9.1 (SAS Institute, Cary, NC, USA). Differences were considered significant at *P *< 0.05.

## Results

### Fucoidan inhibits the growth of HT-29 and HCT116 cells

**Figure 1:**
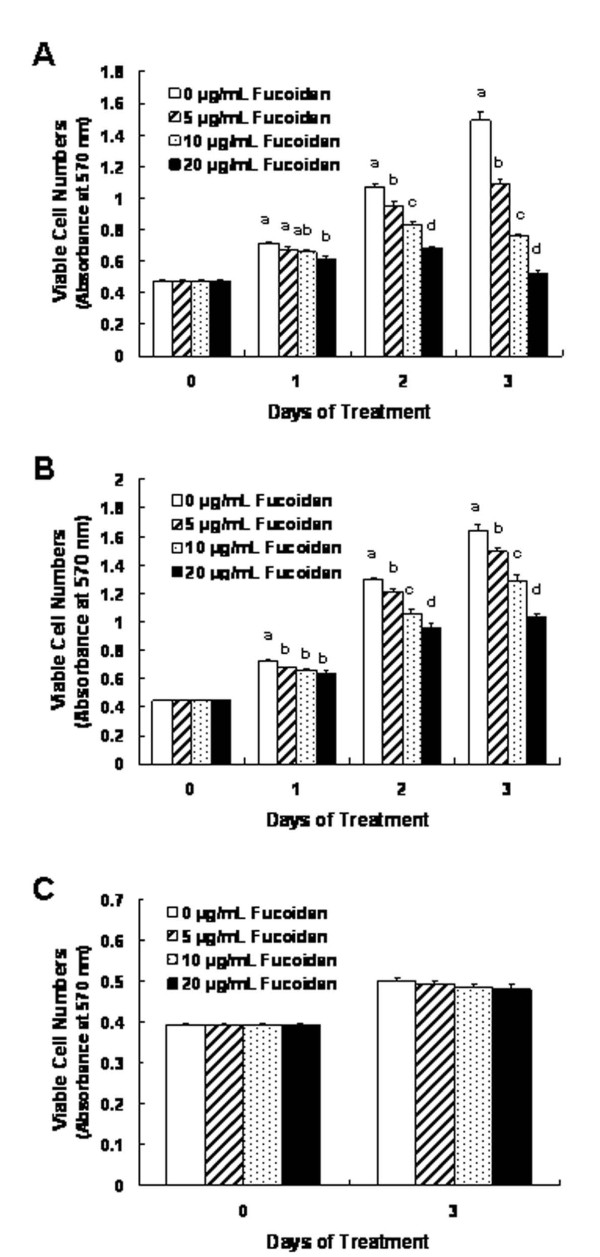
**Fucoidan reduces the viability of HT-29 and HCT116 cells**. HT-29 **(A)**, HCT116 **(B) **and FHC **(C) **cells were plated in 24-well plates at a density of 50,000 cells/well with DMEM/F12 supplemented with 10% FBS. One day later, the monolayers were serum-deprived with DMEM/F12 supplemented with 1% charcoal-stripped FBS (serum-deprivation medium) for 24 h. Following serum deprivation, the cells were incubated in serum-deprivation medium containing 0 - 20 μg/mL of fucoidan. Viable cell numbers were estimated via MTT assays. Each bar represents the mean ± SEM (n = 6). Means at a time without a common letter differ, *P *< 0.05.

We initially assessed the effects of different concentrations (5, 10, 20 μg/mL) of fucoidan on the growth of HT-29 and HCT116 cells by measuring the viable cell numbers via MTT assays. In HT-29 cells, fucoidan reduced the numbers of viable cells in a dose-dependent fashion, with a 64.9 ± 1.5% reduction in cell numbers noted 72 h after the addition of 20 μg/mL (Figure 1A). Fucoidan also inhibited the growth of HCT116 cells. However, the degree of inhibition was smaller in HCT116 cells than was noted with the HT-29 cells. The treatment of HCT116 cells with 20 μg/mL of fucoidan for 72 h resulted in a 36.7 ± 2.0% reduction in the viable cell numbers (Figure 1B). Additionally, we conducted a similar experiment utilizing FHC human normal colon epithelial cells in an effort to determine whether or not fucoidan exerts toxic effects on normal colonocytes. The same concentrations of fucoidan exerted no detectable effects on the viability of FHC cells (Figure 1C).

### Fucoidan induces apoptosis of HT-29 and HCT116 cells

**Figure 2:**
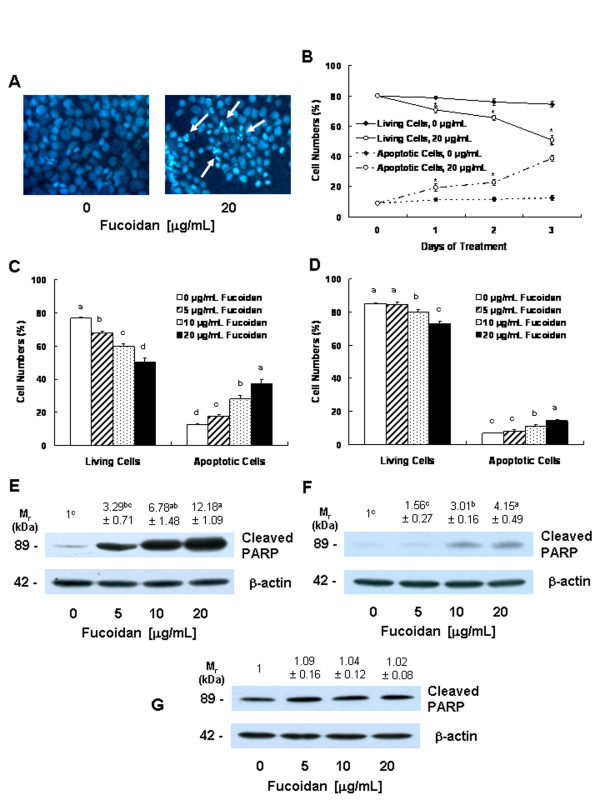
**Fucoidan induces apoptosis of HT-29 and HCT116 cells**. **(A) **HT-29 cells were plated on cell culture coverslips at 50,000 cells/cover and treated for 72 h with fucoidan, as shown in Figure 1. The cells were stained with Hoechst 33258 and the microphotograph images are representative of three independent experiments. Magnification, × 200. **(B) **HT-29 cells were treated with or without 20 μg/mL of fucoidan for the indicated periods as described in Figure 1. HT-29 **(C) **and HCT116 **(D) **cells were treated with various concentrations of fucoidan for 72 h. (**B, C, D**) The cells were trypsinzed, stained with 7-amino-actinomycin D and annexin V, and then analyzed via flow cytometry. The numbers of living cells and early apoptotic cells are expressed as percentages of the total cell number. Results are expressed as the means ± SEM (n = 6). HT-29 **(E)**, HCT116 **(F)**, and FHC **(G) **cells were plated in 100 mm dishes and treated with various concentrations of fucoidan for 60 h, as described in Figure 1. The cell lysates were analyzed via Western blotting with the indicated antibodies. A photograph of chemiluminescent detection of the blots, which were representative of three independent experiments, are provided. The relative abundance of each band to its own β-actin was quantified, and the control levels were set to 1. The adjusted mean ± SEM (n = 3) of each band is shown above each blot. (B) *Significantly different from 0 μg/mL of fucoidan, *P *< 0.05. **(C, D, E, F) **Means without a common letter differ, *P *< 0.05.

In order to determine whether the fucoidan-induced reduction in cell viability was attributable to the induction of apoptosis, we stained the HT-29 cells with Hoechst 33258 dye. The treatment of HT-29 cells with 20 μg/mL of fucoidan resulted in the induction of chromatin condensation and fragmentation, which could be visualized as an intense pycnotic bluish-white fluorescence within the cell nuclei (Figure 2A). We subsequently estimated the numbers of apoptotic cells by staining the cells with Annexin V and 7-AAD, followed by flow cytometry. In HT-29 cells, the proportions of apoptotic, Annexin V-positive/7-AAD-negative cells increased in a time-dependent manner in cells that had been treated with 20 μg/mL of fucoidan (Figure 2B). Additionally, a concentration-dependent increase in the proportions of apoptotic cells was noted after the cells were treated for 72 h with increasing concentrations of fucoidan (Figure 2C). In HCT116 cells, the proportions of apoptotic cells were increased significantly by treatment with 10 μg/mL of fucoidan. However, the proportions of apoptotic cell numbers were lower in HCT116 cells than in HT-29 cells (Figure 2D).

Furthermore, fucoidan treatment resulted in increases in the levels of cleaved PARP in both HT-29 (Figure 2E) and HCT116 cells (Figure 2F). Fucoidan exerted no detectable effects on PARP cleavage in FHC cells (Figure 2G).

### Fucoidan increases the activation of caspases, but reduces the protein levels of IAPs

**Figure 3:**
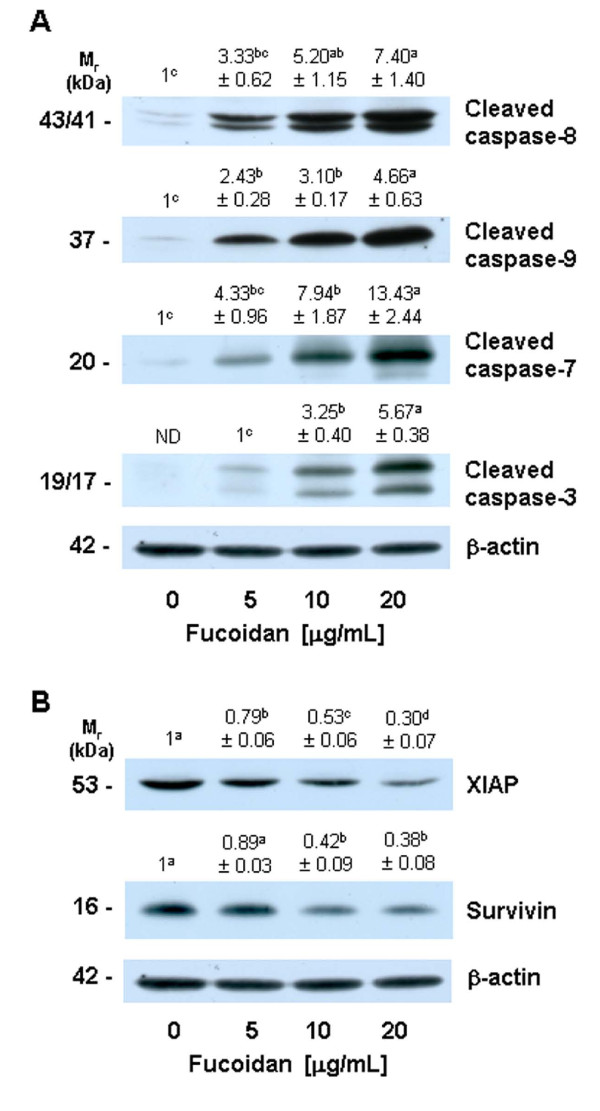
**Fucoidan increases the levels of cleaved caspases and reduces the levels of the inhibitor of apoptosis proteins in HT-29 cells**. Cells were treated with various concentrations of fucoidan for 60 h, as shown in Figure 1. Cell lysates were analyzed via Western blotting with the indicated antibodies. Photographs of chemiluminescent detection of the blots, which were representative of three independent experiments, are shown. The relative abundance of each band to its own β-actin was quantified, and the control levels (0 μg/mL fucoidan) were set to 1 except for caspase-3, for which 1 is set to 5 μg/mL of fucoidan. The adjusted means ± SEM (n = 3) are shown above each blot. Means without a common letter differ, *P *< 0.05.

Caspases are central effectors of apoptosis [24]. As a first step in identifying the mechanisms responsible for fucoidan-induced apoptosis, we attempted to determine whether or not fucoidan activates caspases, via Western blotting using antibodies that detect the cleaved forms of the enzymes. Fucoidan treatment induced concentration-dependent increases in the protein levels of cleaved caspases-8, -9, -7, and -3 (Figure 3A).

IAPs block apoptosis by binding to and inhibiting caspases [32], as well as by neutralizing Smac/Diablo [33]. We performed Western blotting of the cell lysates in order to determine whether or not fucoidan treatment would reduce levels of survivin and XIAP. The levels of XIAP protein were reduced significantly by treatment with increasing concentrations of fucoidan. Additionally, fucoidan at a concentration of 10 μg/mL effectively reduced the levels of survivin protein (Figure 3B).

### Fucoidan increases mitochondrial membrane permeability and the release of cytochrome c and Smac/Diablo from the mitochondria

**Figure 4:**
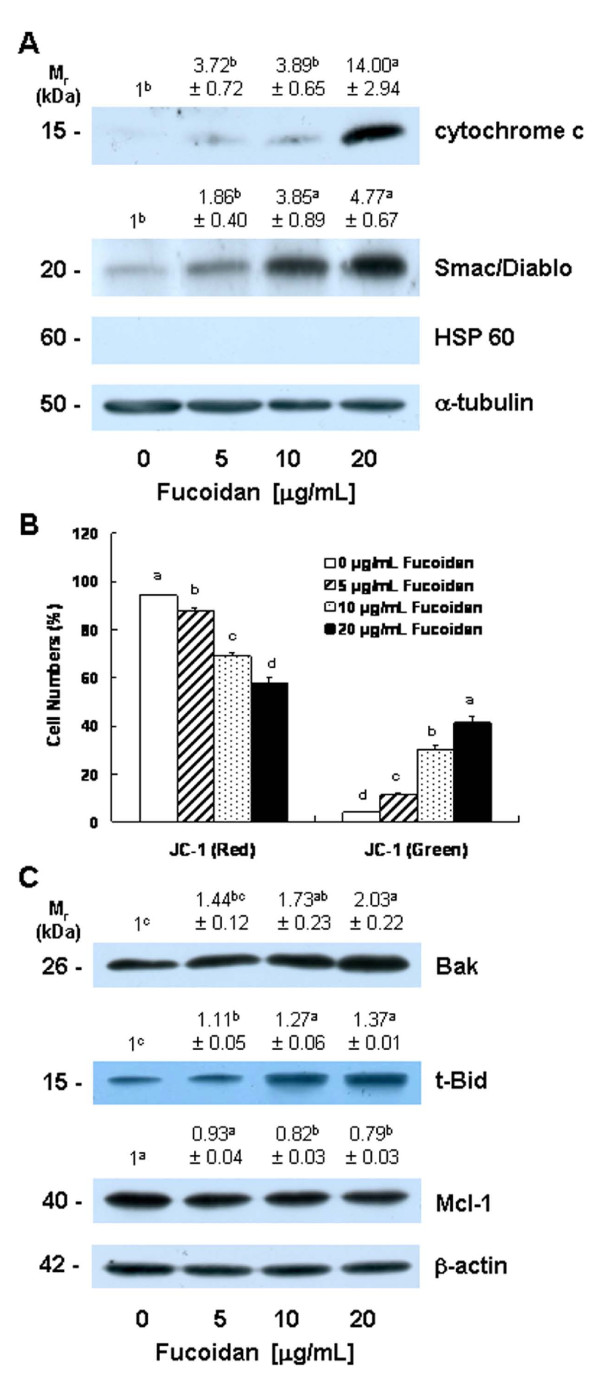
**Fucoidan induces mitochondrial membrane depolarization, increases the release of cytochrome c and Smac/Diablo from the mitochondria, and alters the levels of the Bcl-2 family proteins in HT-29 cells**. **(A) **Cells were treated with various concentrations of fucoidan for 48 h as described in Figure 1, then subjected to subcellular fractionation. The resultant cytosolic fractions were analyzed via Western blotting with the indicated antibodies. Photographs of chemiluminescent detection of the blots, which were representative of three independent experiments, are shown. The relative abundance of each band to its own α-tubulin was quantified, and the control levels were set to 1. The adjusted mean ± SEM (n = 3) of each band is shown above each blot. **(B) **Cells were treated with fucoidan for 48 h. Cells were loaded with JC-1 and then analyzed via flow cytometry. The numbers of cells with normal polarized mitochondrial membranes (red) and with depolarized mitochondrial membranes (green) are expressed as a percentage of the total cell number. Each bar represents the mean ± SEM (n = 6). **(C) **Cells were treated for 36 h with various concentrations of fucoidan as described in Figure 1. Cell lysates were analyzed via Western blotting with the indicated antibodies. Photographs of chemiluminescent detection of the blots, which were representative of three independent experiments, are provided. The relative abundance of each band to its own β-actin was quantified, and the control levels were set to 1. The adjusted means ± SEM (n = 3) of each band are shown above each blot. (**A, B, C**) Means without a common letter differ, *P *< 0.05.

Cytosolic cytochrome c and Smac/Diablo released from the mitochondria promote the activation of caspase-9 [34,35]. Because fucoidan induced the activation of caspase-9, we subsequently attempted to determine whether or not fucoidan treatment induces the release of cytochrome c and Smac/Diablo from the mitochondria. Fucoidan treatment significantly increased levels of cytochrome c and Smac/Diablo in the cytoplasm (Figure 4A). Because fucoidan treatment induced the release of cytochrome c and Smac/Diablo from the mitochondria, we subsequently estimated mitochondrial membrane permeability via JC-1 staining followed by flow cytometry. Fucoidan treatment caused a reduction in the number of cells with intact mitochondria (red-positive and green-negative) and increased the number of cells with depolarized mitochondrial membranes (green-positive and red-negative) in a concentration-dependent manner (Figure 4B).

### Fucoidan alters the levels of the Bcl-2 family proteins

Bcl-2 family proteins play critical roles in the regulation of apoptosis via the control of mitochondrial membrane permeability and the release of cytochrome c and/or Smac/Diablo [36]. Because the permeability of mitochondrial membrane and the release of cytochrome c and/or Smac/Diablo from mitochondria were found to be increased in the fucoidan-treated cells, we subsequently attempted to determine whether or not fucoidan treatment induces changes in the levels of the Bcl-2 family proteins. Fucoidan induced a significant increase in the protein levels of Bak and truncated Bid (t-Bid), the active form of Bid. By way of contrast, Mcl-1 levels were reduced in the fucoidan-treated cells (Figure 4C). The levels of Bcl-2, Bcl-xL, Bax, Bad, Bim, and Bik were not affected by fucoidan treatment (data not shown).

### Fucoidan increases the protein levels of death receptors

**Figure 5:**
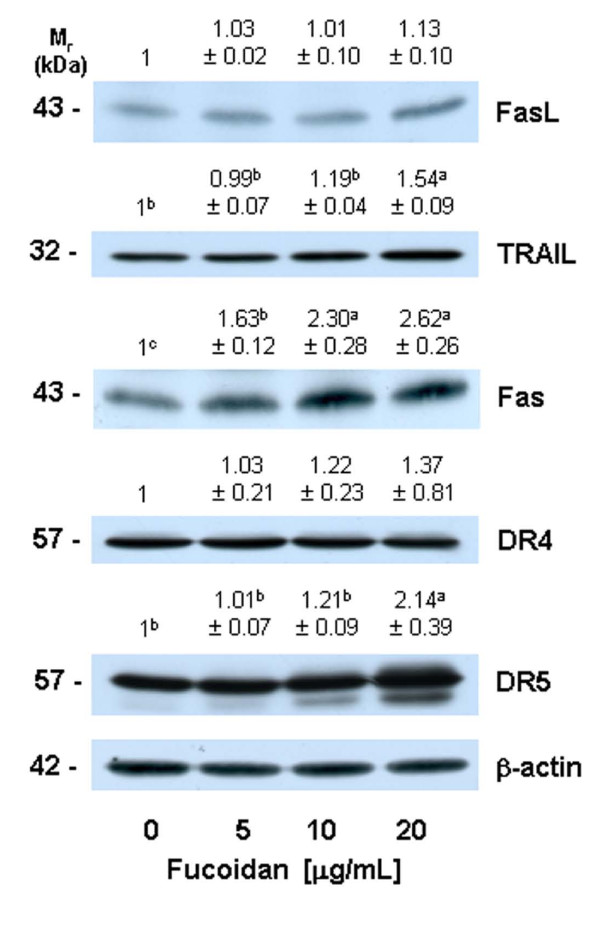
**Fucoidan increases the protein levels of cell death receptors and their ligands in HT-29 cells**. Cells were treated for 36 h with various concentrations of fucoidan, as described in Figure 1. Cell lysates were analyzed via Western blotting with the indicated antibodies. Photographs of chemiluminescent detection of the blots, which were representative of three independent experiments, are shown. The relative abundance of each band to its own β-actin was quantified, and the control levels were set to 1. The adjusted means ± SEM (n = 3) of each band are shown above each blot. Means without a common letter differ, *P *< 0.05.

Because we noted that fucoidan induced an increase in the activation of caspase-8 (Figure 3), we subsequently attempted to determine whether or not fucoidan increases the levels of death receptors and membrane-bound death receptor ligands. Fucoidan treatment resulted in a significant increase in the levels of Fas, DR5, and TRAIL, but exerted no statistically significant effects on the levels of FasL and DR4 (Figure 5).

### The inhibitors of caspase-8 and caspase-9 mitigate fucoidan-induced apoptosis

**Figure 6:**
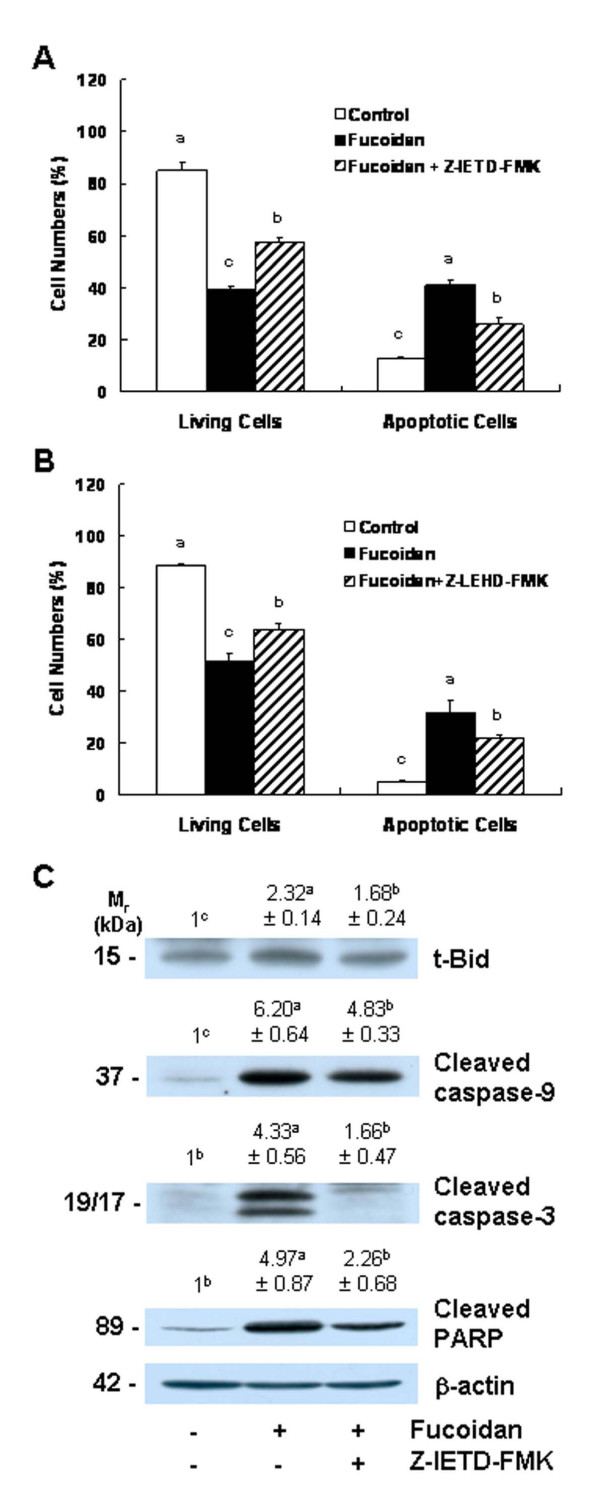
**Caspase inhibitors suppress fucoidan-induced apoptosis in HT-29 cells**. Cells were incubated in the absence or presence of 20 μmol/L of caspase-8 inhibitor (Z-IETD-FMK) **(A) **or caspase-9 inhibitor (Z-LEHD-FMK) **(B) **4 h prior to 48 h of treatment with 20 μg/mL of fucoidan. Cells were stained with 7-amino-actinomycin D and annexin V, then analyzed via flow cytometry. The numbers of living cells and early apoptotic cells are expressed as a percentage of the total cell number. Each bar represents the mean ± SEM (n = 6). **(C) **Cells were treated with the caspase-8 inhibitor (Z-IETD-FMK) and then 20 μg/mL of fucoidan, as described in (A). Total cell lysates were analyzed via Western blotting with the indicated antibodies. Photographs of chemiluminescent detection of the blots, which were representative of three independent experiments, are shown. The relative abundance of each band to its own β-actin was quantified, and the control levels were set to 1. The adjusted means ± SEM (n = 3) of each band are shown above each blot. Means without a common letter differ, *P *< 0.05.

Because caspase-8 and caspase-9 were activated in the fucoidan-treated cells, we attempted to determine, using caspase-8 and caspase-9 inhibitors, whether the inhibition of these caspases would reduce fucoidan-induced apoptosis. The pretreatment of cells with the caspase-8 inhibitor Z-IETD-FMK or the caspase-9 inhibitor Z-LEHD-FMK prior to fucoidan treatment induced a reduction in fucoidan-induced reductions in viable cell numbers and increases in the numbers of apoptotic cells (Figures 6A and 6B). The results of Western blot analyses demonstrated that caspase-8 inhibitors significantly reduced fucoidan-induced increases in the levels of t-Bid. Fucoidan-induced caspase-9 cleavage was moderately, but significantly, suppressed when the cells were pretreated with caspase-8 inhibitor. Caspase-8 inhibitor treatment induced a marked reduction in the fucoidan-induced cleavage of caspase-3 and PARP (Figure 6C).

## Discussion

Dietary habits can affect the development of colorectal cancer [37], and the identification of food components with the ability to prevent the tumorigenic process may facilitate the development of effective agents for the prevention of colon cancer. An ideal chemopreventive agent should be highly effective at multiple sites, orally consumable, minimally toxic or non-toxic, and should employ previously established mechanisms of action [38]. Fucoidan originates from many varieties of edible brown seaweed, and these brown seaweeds have been traditionally consumed in Asia. Fucoidan cannot be hydrolyzed by digestive enzymes in the human small intestine [39], and the consumption of this compound can result in an increase in the concentration of luminal fucoidan within the large intestine. Therefore, fucoidan may prove to be an excellent candidate agent for the prevention of colon carcinogenesis, provided that it exerts cancer-preventive effects in the colon.

The results of previous *in vitro *and *in vivo *studies have shown that fucoidan exerts anti-cancer effects, including the suppression of growth [15-17,19,20,40], metastasis [17,18,21], and angiogenesis [21] in a variety of cancer cells. To the best of our (admittedly limited) knowledge, the effects of fucoidan on colon cancer have yet to be elucidated in detail, with the notable exception of the study of Hyun *et al. *[41], in which fucoidan was determined to induce apoptosis in HCT-15 human colon cancer cells at a concentration of 100 μg/mL. In this study, we noted that fucoidan effectively inhibited the growth of HT-29 and HCT116 cells at concentrations between 5 - 20 μg/mL (Figure 1A). Additionally, identical concentrations of fucoidan exerted no effects on the growth of FHC human normal colon epithelial cells (Figure 1B).

Apoptosis is one of the most prevalent pathways through which chemopreventive/chemotherapeutic agents can inhibit the overall growth of cancer cells. Fucoidan has been shown previously to induce apoptosis in human lymphoma HS-Sultan cells [20], human leukemia U937 cells [40], and MCF-7 human breast cancer cells [42]. In this study, we noted that low concentrations of fucoidan (5 - 20 μg/mL) induced the apoptosis of HT-29 cells in a dose-dependent and time-dependent manner (Figure 2). We also noted that fucoidan induced the apoptosis of HCT116 cells. However, the degree of response to fucoidan was smaller in HCT116 cells than in HT-29 cells. Hyun *et al. *[41] previously reported that high concentrations of fucoidan (100 μg/mL) induced apoptosis in HCT-15 cells. These observations suggest that fucoidan induces apoptosis in human colon cancer cells, but that the efficacy of fucoidan in inducing apoptosis varies among different types of colon cancer cells. The potent *in vitro *efficacy of fucoidan in colon cancer cells indicates that fucoidan may potentially prove useful in the prevention of colon carcinoma. However, it remains to be determined whether or not fucoidan suppresses the development of colon cancer in both animal cancer models and humans. Additionally, it will also be necessary to determine why the degree of response to fucoidan varies among different types of colon cancer cells.

Chemopreventive/chemotherapeutic agents induce apoptosis in a variety of cancer cells via a variety of mechanisms. Aisa *et al. *[20] reported previously that fucoidan induces apoptosis via the activation of caspase-3 and downregulation of the ERK pathway in human HS-Sultan cells. Fucoidan has been shown to induce apoptosis in MCF-7 cells via a caspase-8-dependent pathway [42]. Additionally, Hyun *et al. *[41] reported that 100 μg/mL of fucoidan induced apoptosis in HCT-15 cells via the activation of caspase-9 and -3 accompanied by changes in Bcl-2 and Bax, as well as changes in the phosphorylation of ERK, p38 kinase, and Akt. In this study, we noted that fucoidan at a concentration of 5 - 20 μg/mL 1) increased the activation of caspases, 2) reduced the protein levels of IAPs, 3) increased mitochondrial membrane permeability and cytochrome c and Smac/Diablo release, 4) increased the levels of Bak and t-Bid but reduced the levels of Mcl-1, and 5) increased the levels of Fas, DR5, and TRAIL in HT-29 human colon cancer cells. We also noted that the inhibitors of caspase-8 and caspase-9 reduced fucoidan-induced apoptosis. The results of this study show that fucoidan induces apoptosis through the activation of caspases via both death receptor-mediated and mitochondria-mediated apoptotic pathways.

Caspases perform critically important roles in the induction of apoptosis. Caspases are classified based on their mode of activation as either initiator or effector caspases. Initiators such as caspase-8 and -9 are referred to as apical caspases, which are activated by a variety of apoptotic signals. Activated initiator caspases can cleave and activate effector caspases such as caspase-3 and caspase-7, which in turn cleave a variety of cellular substrates, most notably PARP. One of the most important functions of PARP is to help repair single-strand DNA nicks; thus, cleaved PARP is a useful marker for apoptosis [24,43]. In this study, we determined that fucoidan induces the activation of caspases-8, -9, -3, and -7 (Figure 3), as well as PARP cleavage (Figure 2D). Additionally, we noted that individual caspase-8 or -9 specific inhibitors induced a reduction in fucoidan-induced apoptosis. These results show that the activation of these caspases is one of the principal mechanisms by which fucoidan induces apoptosis.

Caspase activation is triggered primarily via two distinct but interconnected pathways--namely, the death receptor- and mitochondria-mediated pathways. In the death receptor-mediated pathway, the binding of death receptor ligands (e.g., FasL and TRAIL) to their specific death receptors (e.g., Fas, DR4 and DR5) located on the plasma membrane induces the activation of caspase-8. Activated caspase-8 directly triggers the activation of downstream caspase-3 and/or cleaves Bid, a BH3-only pro-apoptotic Bcl-2 family protein. Upon cleavage, t-Bid translocates to the mitochondria, where it enhances the permeability of the mitochondrial membrane, and subsequently induces cytochrome c release and caspase-9 activation [24]. We determined that fucoidan treatment induced an increase in the levels of Fas, TRAIL, and DR5 proteins (Figure 5). Caspase-8 and t-Bid levels were also shown to have increased in the fucoidan-treated cells (Figure 3 and 4). Additionally, we noted that the caspase-8 inhibitor, Z-IETD-FMK, effectively mitigates fucoidan-induced apoptosis and PARP cleavage. Furthermore, this inhibitor was shown to reduce the fucoidan-induced cleavage of Bid, caspase-9, and caspase-3 (Figure 6). These findings demonstrate that the activation of caspase-8 contributes to the activation of caspase-3 via both the direct activation of this enzyme and via the activation of caspase-9 by Bid cleavage, in fucoidan-treated cells. Collectively, our findings demonstrate that the activation of the death receptor-mediated pathway is involved in fucoidan-induced apoptosis in HT-29 cells, via both the direct and indirect activation of caspase-3.

In the mitochondria-mediated pathway, apoptotic stimuli enhance the permeability of the outer mitochondrial membranes and the subsequent release into the cytoplasm of pro-apoptotic factors, including cytochrome c and Smac/Diablo. Cytosolic cytochrome c subsequently binds to apoptosis protease-activating factor 1 (Apaf-1) and inactive procaspase-9 to form an apoptosome, thereby resulting in caspase-9 activation. Activated caspase-9, in turn, triggers the subsequent cleavage of caspases-3 and -7 [34,44]. In addition to demonstrating that fucoidan induces caspases-3 and -7, we determined that fucoidan increased mitochondrial membrane permeability, the release of cytochrome c from mitochondria, and the activation of caspase-9 (Figures 3 and 4). The activation of the mitochondria-mediated pathway results in the release of Smac/Diablo, which confiscates the IAP obstruction of caspase activation [45]. IAPs, including survivin and XIAP, function by binding to and inhibiting several caspases, such as caspase-9 [33]. In this study, we determined that fucoidan treatment increased the release of Smac/Diablo from the mitochondria (Figure 4), but reduced survivin and XIAP levels in HT-29 cells (Figure 3). Our results showed that both the increases in the release of cytochrome c and Smac/Diablo and the downregulation of IAPs were involved in the fucoidan-induced regulation of caspase-9 activity and apoptosis in HT-29 cells.

The permeability of the mitochondrial membrane is regulated precisely by the Bcl-2 family proteins. Anti- or pro-apoptotic Bcl-2 family proteins reside within the cytoplasm or on the outer membranes of the mitochondria. In response to apoptotic stimuli, these proteins form either homo- or hetero-dimers, and then appear to perform distinct functions in the regulation of mitochondrial membrane permeability [46,47]. In this study, fucoidan was shown to affect the levels of Bcl-2 family proteins. Fucoidan treatment increased the levels of pro-apoptotic Bak and t-Bid, but reduced the levels of anti-apoptotic Mcl-1 (Figure 4). The findings of this study demonstrate that the alteration in Bcl-2 family proteins contributed to an increase in mitochondrial membrane permeability and cytochrome c and Smac/Diablo release, and subsequent caspase-9 activation in fucoidan-treated HT-29 cells.

## Conclusion

The results of this study showed that fucoidan inhibits growth and induces apoptosis in HT-29 human colon cancer cells, and this effect is mediated by the activation of caspases. The findings of the present study show that fucoidan activates caspases via both the death receptor-mediated and mitochondria-mediated apoptotic pathways. This study provides a molecular basis for using fucoidan as a potential apoptosis-inducing agent. Thus, studies should be conducted in the future to evaluate the potential of fucoidan as a colon cancer-preventive agent in experimental animal models and in humans.

## Abbreviations

7-AAD: 7-amino-actinomycin D; DMEM/F-12: Dulbecco's Modified Eagle's Medium/Ham's F-12 nutrient mixture; DR: death receptor; FasL: Fas ligand; FBS: fetal bovine serum; HRP: horse-radish peroxidase; HSP: heat shock protein; IAP: inhibitor of apoptosis protein: JC-1,5,5',6,6'-tetrachloro-1,1',3,3'-tetraethyl-imidacarbocyanine iodide; MTT: 3-[4,5-dimethylthiazol-2-yl]-2,5-diphenyltetrazolium bromide; PARP: poly (ADP-ribose) polymerase; t-Bid: truncated Bid; TRAIL: tumor necrosis factor-related apoptosis-inducing ligand; XIAP: X-linked inhibitor of apoptosis protein

## Competing interests

The authors declare that they have no competing interests.

## Authors' contributions

EJK, JYL, and JHYP designed this project. EJK performed the overall biochemical analysis and wrote the first draft of the manuscript. SYP performed Hoechst staining. JHYP directed the overall study and revised the manuscript. All authors contributed to the discussion of the data, and read and approved the final manuscript.

## Pre-publication history

The pre-publication history for this paper can be accessed here:

http://www.biomedcentral.com/1471-230X/10/96/prepub
